# Supplementary material for: MetaRibo-Seq measures translation in microbiomes
Source: Nat Commun. 2020 Jun 29;11:3268. doi: 10.1038/s41467-020-17081-z (PMC7324362; doi:10.1038/s41467-020-17081-z)
Supplement: Supplementary file 10 — Supplementary Data 7 [file 41467_2020_17081_MOESM10_ESM.zip › File2/Confidence_High_Taxonomy/292402_out.krona.html]

Javascript must be enabled to view this page.

members
magnitude
magnitudeUnassigned
count
unassigned
taxon
rank

292402\_out

35

2
35
superkingdom

phylum
35
976

200643
class
35

order
35
171549

2005525
6
family

6
genus
375288


SRS058070\_contig\_number\_contig-100\_340.176070SRS893252\_contig\_number\_14796
1263094
species
2


SRS018836\_contig\_number\_contig-100\_362.155849SRS019968\_contig\_number\_16633SRS054352\_contig\_number\_19919SRS147271\_contig\_number\_37897
46503
4
species

815
29
family

816
29
genus


SRS148196\_contig\_number\_15293SRS148196\_contig\_number\_35193
1929999
2
species

species
1
477666

SRS077552\_contig\_number\_27954


SRS011405\_contig\_number\_9301SRS013940\_contig\_number\_20998SRS018623\_contig\_number\_20182
species
3
329854


SRS051882\_contig\_number\_10256
1
species
1263054


SRS017307\_contig\_number\_27032SRS018623\_contig\_number\_17423SRS048164\_contig\_number\_30087SRS053335\_contig\_number\_10007SRS053335\_contig\_number\_15489SRS063985\_contig\_number\_30517SRS075821\_contig\_number\_14669SRS098571\_contig\_number\_40200SRS098571\_contig\_number\_45328SRS144362\_contig\_number\_11463SRS148159\_contig\_number\_31518SRS148159\_contig\_number\_34794SRS148319\_contig\_number\_13457SRS893369\_contig\_number\_contig-100\_1358.77941
674529
species
14


SRS143523\_contig\_number\_26102
species
1
469590


SRS019445\_contig\_number\_13638SRS046369\_contig\_number\_contig-100\_133.87420SRS148159\_contig\_number\_33212
3
species
28116


SRS015217\_contig\_number\_7905SRS017701\_contig\_number\_12449SRS064973\_contig\_number\_1951SRS104636\_contig\_number\_27669
species
4
818
